# The role of TIPE2 in hemorrhagic shock-induced acute lung injury

**DOI:** 10.1590/acb410326

**Published:** 2026-01-16

**Authors:** Yu-Ying Rong, Yuan-Wei Li, Shi-Ying Yang, Shu-Yan Liu, Yue-Hong Dong, Hui-Bo Du, Xiu Xu, Zi-Gang Zhao, Li-Na Jiang

**Affiliations:** 1 Hebei North University – College of Laboratory Medicine – Department of Immunology – Zhangjiakou – China.; 2 Hebei North University – Institute of Microcirculation – Zhangjiakou – China.; 3 Hebei Key Laboratory of Critical Disease Mechanism and Intervention – Zhangjiakou – China.; 4 Key Laboratory of Microcirculation and Shock – Zhangjiakou – China.; 5 Zhangjiakou University – Zhangjiakou – China.

**Keywords:** Shock, Hemorrhagic, Acute Lung Injury, Inflammation

## Abstract

**Purpose:**

Acute lung injury is the most severe complication of hemorrhagic shock and closely correlates with the mortality rate of hemorrhagic shock. TIPE2 is a critical regulator of inflammation and is implicated in the pathogenesis of various inflammatory diseases. However, its role in hemorrhagic shock-induced acute lung injury is unclear, and the underlying mechanisms remain to be elucidated. Therefore, the purpose of this study was to investigate the role of TIPE2 in hemorrhagic shock-induced acute lung injury and its underlying mechanisms.

**Methods:**

C57BL/6J and TIPE2 knockout mice were used to establish hemorrhagic shock model, with a sham surgery as the control. The pulmonary ventilation function was evaluated using in-vivo testing system. Blood gas analysis was conducted to evaluate changes in blood oxygen level, reflecting the body’s acid-base balance. Hematoxylin and eosin staining facilitated the observation of lesion progression in pulmonary tissue. The expression levels of TIPE2, myeloperoxidase, and citrullinated histone in lung tissues were determined by Western blotting, whereas the levels of tumor necrosis factor-α and IFN-γ in bronchoalveolar lavage fluid were quantified by enzyme-linked immunosorbent assay to evaluate the levels of key inflammatory mediators. VE-cadherin and E-cadherin expression in lung tissues were assessed by Western blotting to indicate changes of lung microvascular and alveolar permeability.

**Results:**

Following hemorrhagic shock, mice developed severe acute lung injury, characterized by impaired lung function, respiratory acidosis, structural damage, and pulmonary edema. This was accompanied by a heightened inflammatory response, evidenced by elevated neutrophil activity and pro-inflammatory cytokines, alongside impaired endothelial and epithelial barrier integrity. Notably, TIPE2 knockout conferred protection against hemorrhagic shock-induced lung injury in mice.

**Conclusion:**

TIPE2 knockout attenuates hemorrhagic shock-induced acute lung injury through mechanisms involving downregulation of inflammatory-associated protein expression, suppression of proinflammatory cytokine release, and restoration of pulmonary barrier permeability.

## Introduction

Hemorrhagic shock (HS) is a systemic pathological condition caused by rapid and substantial blood loss, leading to ischemia, hypoxia, and inadequate organ perfusion. Such dysfunction may progress to multiple organ failure and septic shock, thereby significantly increasing mortality risk^1^^,^ 2.

The lung serves as a critical immune organ and the primary site for respiratory exchange^3^. Ischemia-reperfusion induces a sharp increase in reactive oxygen species that causes acute lung injury (ALI)^4^, which is characterized by the activation and aggregation of neutrophils, release of inflammatory cytokines, severe damage of pulmonary vascular endothelial cells, and increased vascular permeability^5^^–^ 7.

Tumor necrosis factor-α-induced protein 8-like 2 (TIPE2), which is predominantly expressed in immune cells, functions as a key modulator of immune homeostasis, which it achieves by orchestrating the balance between pro- and anti-inflammatory pathways via the bidirectional regulation of cytokine signaling networks. Elevated TIPE2 expression in dendritic cells following HS correlates with systemic immunosuppression; by contrast, reduced TIPE2 levels contribute to reversing the immuno-suppressed state^8^. However, the role of TIPE2 in post-HS-induced ALI remains unclear at present.

In this study, we hypothesized that TIPE2 plays a critical role in the development of HS-induced ALI. To test this hypothesis, we investigated the effects of TIPE2 deletion on pulmonary inflammation and injury in a murine model of HS.

## Methods

### Animals

Male C57BL/6J mice aged 8–10 weeks old, specific pathogen free (SPF) grade, were purchased from SiPeiFu Biotechnology Ltd. (Laboratory Animal License Number: SCXK 2019-0010). Male TIPE2^-/-^ mice of SPF grade, aged 8–10 weeks old, were obtained from Beijing Yaokang Technology Ltd. (Laboratory Animal License Number: SCXK 2023-0008). All experimental mice were maintained at Hebei North University’s Microcirculation Research Institute Animal Facility under controlled environmental conditions (23 ± 2°C, 50 ± 10% humidity). Following a 12-hour fasting period with free access to water, the mice were subsequently subjected to the experiment.

The experimental protocol was approved by the Ethics Committee of Hebei North University and complied with national guidelines for laboratory animals welfare and ethical use.

### Hemorrhagic shock model and experimental grouping

Twenty male wild-type mice and 20 TIPE2-knockout mice, aged 8–10 weeks old, were randomly divided into four groups, with 10 mice in each group:

- Wild-type sham surgery (WT-sham) group;
- Wild-type HS (WT-shock) group;
- TIPE2-knockout sham surgery group (TIPE2-/- -sham);
- TIPE2-knockout HS (TIPE2^-/-^- shock) group.

The HS model was induced in accordance with the established laboratory protocol^9^. The mice were anesthetized with 3% isoflurane inhalation for induction to reach a light anesthesia level, followed by administered general anesthesia with 1% sodium pentobarbital. After thorough exposure and disinfection of bilateral inguinal skin, surgery involving catheterization of bilateral femoral arteries was performed under a microscope on mice. One side of the femoral artery was linked to a multi-channel biological signal acquisition system to monitor the mean arterial pressure in real-time, whereas the other side was attached to a syringe and affixed to a microinfusion pump (from American New Times Company) for withdrawing blood and fluid resuscitation, when the mean arterial pressure reached and kept (40 ± 2) mmHg for 90 min. Then, resuscitation with blood and an equal volume of Ringer’s solution were applied in 30 min at a controlled rate for fluid. The sham group underwent identical surgical procedures as the shock group, except blood loss. Tissue samples were collected from each groups following a 3-h observation period for subsequent experiments.

### Pulmonary function measurement

After a 3-h observation period of each experimental group, mice were anesthetized to expose their neck skin. Subsequently, controlled mechanical ventilation was delivered through the indwelling endotracheal cannula. The pulmonary function was tested by a pulmonary function testing system (PFT, DSI, Buxco Research Systems, United States of America). The inspiratory capacity (IC), forced vital capacity (FVC), forced expiratory volume in 100 ms (FEV100), functional residual capacity (FRC), dynamic lung compliance (Cdyn), peak expiratory flow (PEF), and pulmonary resistance index (RI) were calculated and recorded.

### Arterial blood gas analysis

Finishing postoperative observations of each experimental group, arterial blood was obtained by puncturing the abdominal aorta with a disposable blood collection needle. Blood gas analysis cards were utilized to examine the levels of PaCO_2_, lactate (Lac), and pH in the mice and computed the oxygenation index (PaO_2_/FiO_2_), then statistical analysis was conducted on the gathered data.

### Lung wet/dry weight ratio

HS-induced ALI leads to pulmonary tissue edema characterized by an elevation in water content^10^. Lung tissues from mice in each experimental group were sterilely collected and weighed using a precise balance, then the documenting wet weight of the lungs was recorded. Lung tissues were desiccated at 65°C for 48 h to determine dry mass, with pulmonary edema severity assessed via wet/dry weight ratio calculation.

### Histopathological lung examination

The lung tissues were immersed in 4% paraformaldehyde 24 h at 4°C, then dehydration, clarification, embedding, sectioning, and staining with hematoxylin and eosin to exam followed. The histological injury scores were determined based on the cumulative score for alveolar edema, alveolar hemorrhage, pulmonary interstitial thickening, and neutrophil infiltration. Each histological feature was assessed on a scale from 0 to 4:

- 0: normal (no damage);
- 1: indicating minimal damage (affecting up to 25% of the field);
- 2: suggesting mild damage (involving 25 to 50% of the field);
- 3: signifying moderate damage (spanning 50 to 75% of the field);
- 4: denoting severe damage (involving over 75% of the field)11.

### Western blotting analysis

Lung tissues from each experimental group were harvested aseptically. The tissues were thoroughly homogenized and sonicated to obtain total protein, which was quantified using BCA kits. SDS-PAGE gel electrophoresis was employed to separate TIPE2, myeloperoxidase (MPO), citrullinated histone (Cit-H3), E-cadherin, and VE-cadherin of the lung tissues of each experimental group. In a setting of steady current, proteins from the gel were moved onto a PVDF membrane and then obstructed using a 5% skim milk solution for 1.5 h. Following this, the membrane was exposed to primary antibodies targeting TIPE2 (1:1,000 dilution), MPO (1:10,000 dilution), Cit-H3 (1:10,000 dilution), E-cadherin (1:1,000 dilution), VE-cadherin (1:1,000 dilution) and glyceraldehyde 3-phosphate dehydrogenase (GAPDH) (1:20,000 dilution), incubated overnight. The next day, the membrane underwent a 1.5-h incubation with an HRP-conjugated secondary antibody (1:10,000 dilution) at room temperature. Finally, images were examined with an Tanon 5800 Multi imager, and the strip optical density was analyzed by ImageJ.

### Enzyme-linked immunosorbent assay analysis

The tracheas of mice were exposed and cut in each experimental group. A gastric tube was inserted in them, and 1 mL pre-cooled physiological saline was aspirated with a 1-mL syringe. The saline was slowly injecting into the lungs, and the mouse thorax was gently massaged for 20 s, to collect the bronchoalveolar lavage fluid (BALF), repeating the process three times to ensure a recovery rate > 75% for the lavage fluid. BALF centrifuged for 10 min (1,200 r/min). The concentrations of tumor necrosis factor (TNF)-α and IFN-γ were analyzed by enzyme-linked immunosorbent assay (ELISA) kits (Wuhan Chundu Biotechnology Co, Ltd, China), drawing a standard curve, calculating the corresponding concentrations of inflammatory factors, followed by statistical analysis to determine differences.

### Statistical analysis

The experiment’s data underwent statistical analysis with GraphPad Prism 9.5.1 and are displayed as mean ± standard deviation. One-way analysis of variance (ANOVA) was utilized to compare data across multiple groups, and a significance threshold of *p* < 0.05 denoted statistical significance.

## Results

### TIPE2 deletion lengthen the survival time of mice following hemorrhagic shock

**Figure 1:**
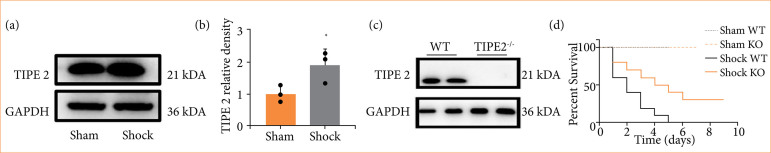
Hemorrhagic shock enhanced the TIPE2 expression of lung tissue and TIPE2 deficiency prolonged the survival time of mice subjected to hemorrhagic shock. **(a)** Characteristic images. **(b)** Changes of TIPE2 from Western blotting analysis. Data are showcased as the mean ± standard deviation (n = 3). *p < 0.05 versus WT sham. **(c)** Identification of mouse gene. **(d)** Bar chart of survival time of mice. Data are presented as mean ± standard deviation (n = 10). *p < 0.05 compared with the sham group.

TIPE2: tumor necrosis factor-α-induced protein 8-like 2; GAPDH: glyceraldehyde 3-phosphate dehydrogenase; WT: wild-type mice. Source: Elaborated by the authors.

Western blotting was utilized to characterize TIPE2 expression in lung tissues of sham group and shock group. The result demonstrated a significant increase of TIPE2 expression of the shock group in comparison with the sham group (Fig. 1a, b). Assessing TIPE2 expression in lung tissues to genotype mice, results indicated that TIPE2^-/-^ mice are suitable for experiments as homozygotes (Fig. 1c). The survival period of mice in each experimental group showed that survival time of TIPE2^-/-^ mice was significantly longer than wild-type (WT)mice followingHS.The results showed that the expression of TIPE2 of mice related to the survival time of mice following hemorrhagic shock (Fig. 1d).

### TIPE2 deletion improved lung respiratory dysfunction and alleviated pulmonary ventilation

**Figure 2:**
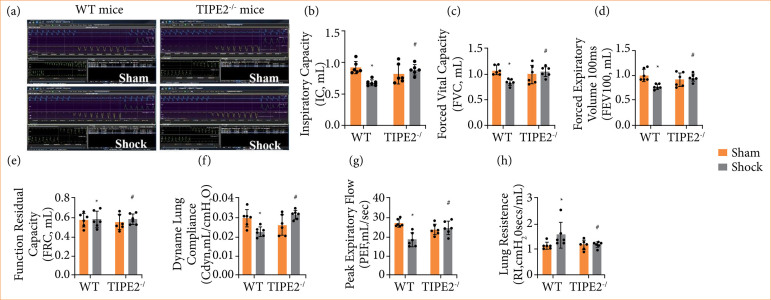
TIPE2 deficiency improved pulmonary respiratory insufficiency of mice following hemorrhagic shock. **(a)** The original curve of respiration. **(b)** Inspiratory capacity. **(d)** Forced vital capacity. **(d)** Forced expiratory volume in 100 ms. **(e)** Functional residual capacity. **(f)** Dynamic lung compliance (Cdyn). **(g)** Peak expiratory flow. **(h)** Pulmonary resistance index. Data are presented as the mean ± standard deviation (n = 6).

TIPE2: tumor necrosis factor-α-induced protein 8-like 2; WT: wild-type mice; *p < 0.05 versus WT sham; ^#^ *p* < 0.05 versus WT shock. Source: Elaborated by the authors.

In order to delve deeper into the impact of TIPE2 on lung injury following HS, the pulmonary function testing system was utilized to evaluate alterations in pulmonary respiratory function across all experimental *in-vivo* groups (Fig. 2a). The findings demonstrated that there were notable decreases in IC (Fig. 2b), FVC (Fig. 2c), FEV100 (Fig. 2d) and PEF (Fig. 2g) in the shock group, along with the decrease in Cdyn (Fig. 2f), when compared it with the sham group (*p* < 0.05). However, there were no significant alteration in FRC (Fig. 2e) and RI (Fig. 2h). The TIPE2 deletion resulted in a marked reversal of these alterations (*p* < 0.05). The results indicated that the expression changes of TIPE2 in mice lung tissues were associated with HS-induced ALI, and removal of TIPE2 may help improve post-HS lung respiratory dysfunction and alleviate pulmonary ventilation.

### TIPE2 deletion is beneficial in alleviating the acidosis and hypoxic state

**Figure 3:**
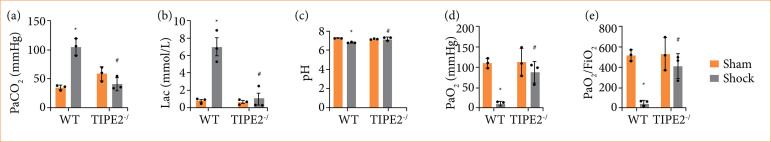
TIPE2 deficiency corrected hypoxia and acidosis of mice following hemorrhagic shock. **(a)** PaCO_2_. **(b)** Lactase (Lac). **(c)** pH. **(d)** PaO_2_. **(e)** PaO_2_/FiO_2_. Data are presented as the mean ± standard deviation (n = 3).

TIPE2: tumor necrosis factor-α-induced protein 8-like 2; WT: wild-type mice; *p < 0.05 *versus* WT sham; #*p* < 0.05 *versus* WT shock. Source: Elaborated by the authors.

Rapid, massive blood loss precipitates hypoxia and severe acidosis in the context of HS^12^, as shown in Fig. 3. pH (Fig. 3a), PaO_2_ (Fig. 3b), and PaO_2_/FiO2 (Fig. 3c) declined (*p* < 0.05), while the arterial carbon dioxide partial pressure (PaCO_2_, Fig. 3d) and Lac (Fig. 3E) rose (*p* < 0.05) in the shock group, compared with the sham group. There was a significant improvement in the acidosis condition, characterized by a substantial increase in pH values (Fig. 3a, *p* < 0.05), PaO_2_ (Fig. 3b, *p* < 0.05), PaO_2_/FiO_2_ (Fig. 3c, *p* < 0.05) and a notable reduction in PaCO_2_ (Fig. 3d, *p* < 0.05) and Lac (Fig. 3e, *p* < 0.05), after deleting TIPE2. The finding suggests that deletion TIPE2 markedly improved the hypoxic and acidotic conditions and pulmonary gas exchange function following HS.

### TIPE2 deletion improved pulmonary morphology and reduced water content

**Figure 4:**
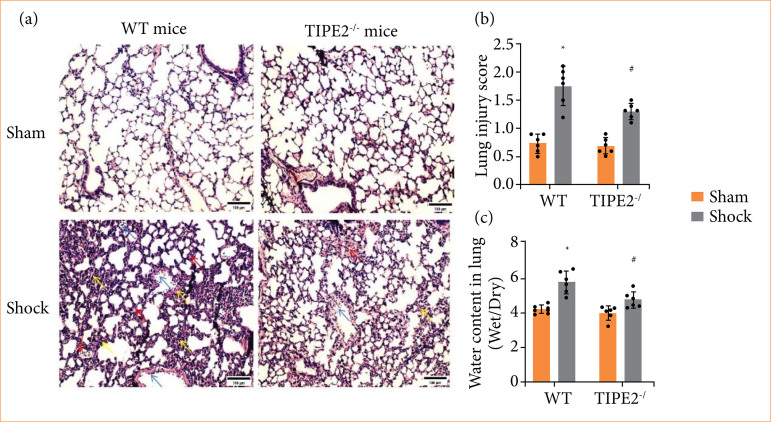
TIPE2 deficiency improved pulmonary morphology and reduced the water content in lungs of mice subjected to hemorrhagic shock. **(a)** Representative images of lung after hematoxylin and eosin staining (scale bar =100 μm). **(b)** Histological lung injury score. **(c)** Wet/dry ratio. Data are presented as the mean ± standard deviation (n = 5).

TIPE2: tumor necrosis factor-α-induced protein 8-like 2; WT: wild-type mice; *p < 0.05 *versus* WT sham; ^#^ *p* < 0.05 *versus* WT shock. Source: Elaborated by the authors.

During ALI, the histopathological features observed in lung tissues encompass alveolar collapse, substantial infiltration of inflammatory cells, as well as interstitial congestion and edema^13^. Upon examination of the lung tissues of mice from different experimental groups using hematoxylin and eosin (HE) staining and lung injury score, it was observed that almost normal structures in sham group, such as the pulmonary tissue exhibited alveolar structures of diverse sizes, filled with air, and characterized by thin septa facilitating gas exchange. In shock group, lung tissues exhibited interstitial congestion, substantial infiltration of inflammatory cells, and additional markers of lung injury (Fig. 4a), and the lung injury score showed a significant increase compared to the sham group (Fig. 4b, *p* < 0.05). Following HS, there was an amelioration in lung tissue pathological damage of TIPE2 knockout mice, compared to the WT shock group, characterized by decreased infiltration of inflammatory cells and mitigation of edema (Fig. 4a) and the lung injury score (Fig. 4b, *p* < 0.05).

Evaluating the pulmonary tissue edema of mice from different experimental groups by calculating the lung wet/dry ratio, there was a notable elevation of wet/dry ratio in the shock group in WT mice, which reduced in removal of TIPE2 mice following HS (*p* < 0.05), as Fig. 4C shows.

### TIPE2 deletion reduced the levels of MPO, Cit-H3, IFN-γ, and TNF-α

MPO is an enzyme present in neutrophils involved in the generation of oxidants to eliminate pathogens. Cit-H3 is a protein associated with inflammation and autoimmune diseases. Elevated expression of them is typically associated with increased neutrophil activity and heightened inflammatory responses.

**Figure 5:**
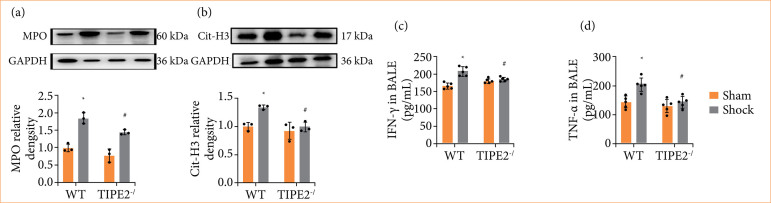
Effects of TIPE2 deficiency on the levels of MPO, Cit-H3, IFN-γ, and TNF-α in the lungs of mice following hemorrhagic shock. (a and b) Characteristic images and changes of MPO and Cit-H3 from Western blotting analysis. Data are showcased as the mean ± standard deviation (n = 3). (c and d) Levels of IFN-γ and TNF-α from the enzyme-linked immunosorbent assay analysis. Data are presented as the mean ± standard deviation (n = 5).

TIPE2: tumor necrosis factor-α-induced protein 8-like 2; MPO: myeloperoxidase; Cit-H3: citrullinated histone; GAPDH: glyceraldehyde 3-phosphate dehydrogenase; TNF: tumor necrosis factor; WT: wild-type mice; *p < 0.05 *versus* WT sham; #*p* < 0.05 *versus* WT shock. Source: Elaborated by the authors.

The results from Fig. 5 revealed that HS enhanced the expression of MPO (Fig. 5a) and Cit-H3 (Fig. 5b) in pulmonary, which were decreased by deleting TIPE2 (*p* < 0.05). IFN-γ and TNF-α, which are commonly regarded as pro-inflammatory cytokines, may promote the onset of inflammatory responses through their elevated expression. IFN-γ (Fig. 5c) and TNF-α (Fig. 5d) levels of BALF were enhanced highly after HS, while the upward trend is reversed by deleting TIPE2 (*p* < 0.05). The results indicated that the expression level of TIPE2 was associated with the extent of inflammatory responses in lung tissues following HS, and deleting TIPE2 significantly alleviated this response.

### TIPE2 deletion alleviated changes of lung permeability

**Figure 6:**
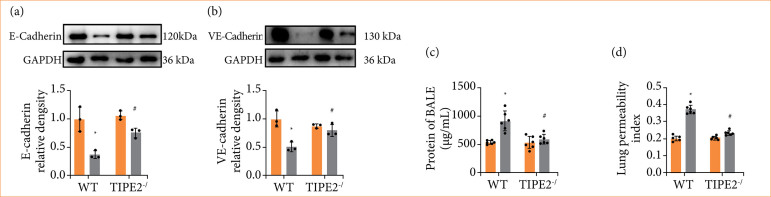
Effects of TIPE2 deficiency on lung permeability following hemorrhagic shock. (a and b) Characteristic images and changes of E-cadherin and VE-cadherin from Western blotting analysis. Data are presented as the mean ± standard deviation (n = 3). **(c)** Protein content of BALF. **(d)** Lung permeability coefficient. Data are presented as the mean ± standard deviation (n = 6).

TIPE2: tumor necrosis factor-α-induced protein 8-like 2; GAPDH: glyceraldehyde 3-phosphate dehydrogenase; BALF: bronchoalveolar lavage fluid; WT: wild-type mice; *p < 0.05 *versus* WT sham; #*p* < 0.05 *versus* WT shock. Source: Elaborated by the authors.

VE-cadherin maintains adhesion among pulmonary vascular endothelial cells, preserving vascular wall integrity. E-cadherin facilitates pulmonary epithelial cell connections, preserving alveolar structure integrity. These two proteins are crucial about the regulation of lung permeability, which reduced expression may alter lung permeability, thereby affecting pulmonary function. The results from Fig. 6 revealed that the expression of VE-cadherin (Fig. 6a) and E-cadherin (Fig. 6b) in pulmonary were reduced significantly following HS, which were enhanced after deleting TIPE2. Protein content in BALF (Fig. 6c) and lung permeability index (LPI) (Fig. 6d) significantly increased following HS, while the changes were reversed by deleting TIPE2. These findings suggested that deleting TIPE2 helped to ameliorate the alterations of lung tissues permeability following HS.

## Discussion

HS leads to systemic hypoperfusion, resulting in impaired oxygen delivery, cellular hypoxia, and additional circumstances. It presents a significant danger to patient well-being. Currently, significant progress has been made in the clinical treatment of hypovolemia. However, due to organ reperfusion injury and immune dysfunction, severe complications can still arise. Among these, ALI is one of the most common and serious complications^14^^,^ 15. ALI is characterized by diffuse pulmonary interstitial and alveolar edema, resulting from damage to pulmonary capillary endothelial cells and alveolar epithelial cells in non-cardiogenic conditions like severe infection, HS, trauma, and burns, leading to acute hypoxemic respiratory failure or respiratory insufficiency^16^^,^ 17. Presently, ALI has emerged as a major contributor to elevated mortality rates in conditions like HS and sepsis. Clinical treatments for ALI still primarily focus on increasing mechanical ventilation and have not identified effective therapeutic targets in terms of immune interventions yet. Therefore, effective therapeutic strategies still need to be found for HS-induced ALI.

TIPE2, a member of the TNFAIP8 family, is primarily expressed by immune cells and serves as a crucial regulatory factor in autoimmune responses^18^. Research has shown that TIPE2 exhibits dual roles in regulating inflammation. On one hand, it suppresses the production of IL-17A by T-cells to alleviate inflammatory responses; on the other hand, it enhances inflammation by promoting the directional migration of T-cells^19^. Additionally, as phosphatidylinositol transfers protein of the TIPE family, TIPE2 functions as a modulator of leukocyte chemotaxis by regulating cell polarization and cytoskeletal dynamics.

Spinal cord slices from TIPE2 knockout mice demonstrated decreased leukocyte infiltration in the experimental autoimmune encephalomyelitis model^20^. In murine tumor models, deletion of TIPE2 enhances the expression of anti-tumor mediators and diminishes the expression of pro-tumor mediators within myeloid-derived suppressor cells, thus hampering tumor growth^21^^–^ 23. Recent research indicated that deleting TIPE2 could dampen the activation of the JAK2/STAT3 signaling pathway, causing a downregulation in the production of pro-inflammatory cytokines (TNF-α, IL-1β, IL-6, and IFN-γ), thereby alleviating the disruption of cell-cell tight junctions induced by pro-inflammatory cytokines^24^^–^ 27. The balance between the anti-inflammatory and pro-inflammatory mediated by TIPE2 might impact the progression of diseases ultimately^19^. Presently, the specific role of TIPE2 in ALI post-HS remains unexplored. The potential therapeutic impact of TIPE2 deletion on HS-induced ALI presents a critical research inquiry.

## Conclusion

This study, based on the relationship between TIPE2 and ALI, investigated the impact of varying expressions of TIPE2 in ALI following HS. The results of this study indicated that during HS, the body is in a state of hypoxia and severe acidosis. *In-vivo* functional lung indicators demonstrated that HS can lead to pulmonary dysfunction in mice, resulting in respiratory distress. Analysis of MPO, Cit-H3, TNF-α, IFN-γ, and the lung wet/dry ratio revealed that HS causes neutrophils aggregation in lungs, triggering intense inflammatory responses, and severe pulmonary tissue edema. However, the hypoxic state and acidosis during HS were observed in TIPE2 deficient mice. This improvement alleviated respiratory dysfunction by reducing pulmonary elastic and airway resistance. Furthermore, lung inflammatory cell infiltration and pulmonary edema were reduced, indicating that the decreased expression of TIPE2 significantly mitigated ALI during HS.

The protective effect of TIPE2 deletion observed appears to contradict reports from other disease contexts in which its deficiency exacerbated injuries. However, growing evidence, including a recent study demonstrating a pro-inflammatory role for TIPE2 in experimental colitis, collectively indicates that its function is not universal but highly context-specific^28^. Thus, the present findings define the detrimental role of TIPE2 in HS-induced ALI, thereby expanding the understanding of its pleiotropic functions.

This study validated from multiple aspects that the absence of TIPE2 confers a protective effect against HS-induced ALI. However, there are still certain limitations: this experiment discovered the protective effect on HS-induced ALI from the perspective of TIPE2 deficiency, but the specific mechanism requires further investigation to provide new directions for its clinical treatment.

## Data Availability

Due to privacy concerns, the datasets from this study are not publicly accessible. However, they can be obtained in anonymized form from the corresponding author upon request.
